# Alcohol and stroke: the splitters win again

**DOI:** 10.1186/s12916-016-0750-z

**Published:** 2016-11-24

**Authors:** Arthur L. Klatsky, H. Nicole Tran

**Affiliations:** 1 Division of Research, Northern California Kaiser Permanente, 2000 Broadway, Oakland, CA 94612 USA; 2 Department of Medicine, Oakland Medical Center, Northern California Kaiser Permanente, Oakland, CA USA

**Keywords:** Stroke, Hemorrhagic stroke, Ischemic stroke, Alcohol drinking, Epidemiology, Risk factors, Non-linear curves, Confounding

## Abstract

Study of the relationships of alcohol drinking and risk of stroke can readily become mired in the labyrinthine interactions of drinking categorizations, non-linear associations, disparate cardiovascular conditions, and the heterogeneous types of stroke. This Commentary discusses the recent article by Larsson et al. (BMC Medicine 14:178, 2016). The authors split their material into separate meta-analyses of subarachnoid hemorrhage, intracerebral hemorrhage, and ischemic stroke, finding disparate alcohol–stroke relationships. Our Commentary pursues the disparity theme, using the lumpers versus splitters paradigm to explore several aspects of this complex area.

Please see related article: http://bmcmedicine.biomedcentral.com/articles/10.1186/s12916-016-0721-4.

## Background

Lumpers and splitters, terms first used by Charles Darwin in 1857 [1], can become opposing factions in any field when there is need to create classifications and assign examples to them [2]. While “lumpers” assign broad categories based on similarities and patterns, “splitters” see important distinctions that have meaningful consequences. The importance of splitting when classifying clinical medical conditions by etiology, severity, or targeted therapy is familiar to health practitioners. Epidemiologists face analogous issues when studying exposures or categorizing covariates, e.g., is the role of cigarette smoking properly examined by looking at “current smokers” versus others, or is it better to split smokers into groups characterized by intensity, duration, and type of cigarette?

Until recent decades, studies of alcohol drinking and health were usually either limited to heavy drinkers or lumped all drinkers together. The possibility of benefit from lighter drinking was raised in 1926 by Pearl’s study of tuberculosis patients [3], where “heavy/steady” drinkers had the highest mortality, followed by “abstainers”, and finally by “moderate” drinkers. Most recent studies confirm this relation to total mortality [4], with the lowered risk in lighter drinkers due preponderantly to a lower coronary artery disease (CAD) risk, an association not reported until 1974 [5]. Pearl’s major contribution was to realize the fallacy in comparing all drinkers to abstainers. Such comparison masks differences between heavy and light/moderate drinkers, either spuriously increasing the apparent risks of lighter drinking or masking the lower risk. His words were memorable, ‘*one cannot judge the role of diet by starvation or excess*.’

Almost all contemporary studies separate light/moderate from heavy drinkers. In determining thresholds for harmful alcohol effects, however, under-reporting of intake creates lumping by placing some heavy drinkers in the light/moderate categories. Since under-reporting is difficult to ascertain, one must conclude that many thresholds for harmful alcohol effects are higher than appears to be the case [6].

The alcohol epidemiology literature has included much debate about whether the optimal referent group should be lifelong abstainers, very light drinkers, or “occasional” drinkers. One point of universal agreement is that use of all abstainers, a group that includes persons that quit drinking because of illness or symptoms (“sick quitters”), might produce data spuriously showing benefit from light/moderate alcohol drinking.

Much interest has arisen about the health effects of non-alcoholic ingredients in beverages, especially phenolic compounds with anti-oxidant properties most abundant in red wines. Efforts to split all drinkers into drinkers of wine, beer, and spirits have run into problems related to overlapping categories and confounding by disparate user traits between persons that drink preponderantly one beverage type.

## Alcohol and cardiovascular conditions

Heavy drinking is associated with increased risk of dilated cardiomyopathy, systemic hypertension, and atrial fibrillation (AF) [3]. Lighter drinking has little or no association with these conditions but carries lower risk of CAD, the most common cardiovascular condition. These conditions not only interact with each other but also with the different types of stroke (Table 1). An example of the complexity represented is AF. This common rhythm disturbance can be a consequence of cardiomyopathy, systemic hypertension, CAD, or other types of heart disease. It can also occur as a direct consequence of heavy alcohol drinking, especially binges (the “Holiday Heart” phenomenon), as a complication of certain non-cardiovascular conditions, or with no evident underlying basis. Stroke from cerebral emboli is the most-feared complication. Thus, usual AF treatment includes anticoagulant therapy, which, in turn, increases risk of cerebral hemorrhage. Performance of a comprehensive analysis of relationships of alcohol drinking to AF needs to split subjects with AF into multiple categories.

**Table 1:** Alcohol and cardiovascular conditions

Cardiovascular Condition	Probable alcohol relationship		Potential impact on stroke risk
	Lighter drinking^a^	Heavier drinking^b^	
Dilated cardio-myopathy [4]	None	Causes a subset	Can cause AF, with higher cardioembolic stroke risk and higher HS risk if on anti-coagulant therapy
Systemic hypertension [4, 12]	Little or none	Probably causal in susceptible persons	A major risk factor – by direct and indirect mechanisms – for all types of stroke
Atherosclerotic coronary disease [4, 5]	Protective	? Less protective, no relationship or increased risk	1) lower risk of AF 2) higher risk of cardioembolic stroke from mural thrombus 3) higher risk of HS if on anti-thrombotic medication
Valvular disease	Data lacking	Data lacking	Multiple conditions; some with higher risk of AF, cardioembolic stroke and higher HS risk if on anti-coagulant therapy
Supra-ventricular arrhythmia [4]	Little or none	Probably a causal factor, especially binges	Includes AF, with higher cardioembolic stroke risk and higher HS risk if on anti-coagulant therapy
Hemorrhagic stroke [8–15]	Unrelated or slightly higher risk	Increased risk	Needs splitting into types – see Table 2
Ischemic stroke [8, 9, 11–14]	Protective – varies with subtypes	Probable higher risk; varies with subtype	Needs splitting into types – see Table 2

*AF* atrial fibrillation, *HS* hemorrhagic stroke

^a^Two or less alcoholic drinks of standard size

^b^Three or more alcoholic drinks of standard size

We hope that the complexity contained in these interactions is conveyed by the brief summary in Table 1; for more detail see Klatsky et al. [4].

## Alcohol and stroke

Stroke is a heterogeneous group of conditions consequent to brain hemorrhage (hemorrhagic stroke; HS) of two types, subarachnoid and intracerebral hemorrhage or ischemic infarction (IS). IS can result from thrombosis in atherosclerotic lesions in large intra-cranial or extracranial blood vessels, embolism from the aorta, carotid vessels, or heart, or atheromatous blockage of small vessels (lacunar infarcts); some are of unclear cause. Risk factors for the different types vary and there are important racial/ethnic disparities. Table 2 presents an attempt to show some of the interactions with alcohol drinking; the intent is primarily to demonstrate how Byzantine this area becomes and why it has been called a “labyrinth” [7].

**Table 2:** Alcohol and stroke

Stroke Type	Probable alcohol relationship		Potential cardiovascular disease interactions
	Lighter drinking^a^	Heavier drinking^b^	
Hemorrhagic stroke			
Subarachnoid hemorrhage [8–15]	Unrelated or slightly higher risk	Increased risk	Risk factors include HTN, smoking, anti-coagulant therapy Noteworthy, but possibly unrelated to other cardiovascular conditions are genetic factors
Intracerebral hemorrhage [8, 9, 11–14]	Unrelated or slightly higher risk	Increased risk	Risk factors include HTN, anti-coagulant therapy, possibly other CAD risk traits
Ischemic stroke			
All ischemic stroke [8, 9, 11–14]	Lower risk	Unrelated or increased risk	It is unclear which ischemic stroke subgroups are involved and/or whether the association is due to direct alcohol effects or to indirect associations secondary to cardiovascular conditions (see Table 1)
Cardioembolic	Need data	Need data	Risk factors include AF, mural thrombi, valve disease Lower by anticoagulant therapy
Artery to artery	Need data	Need data	Aorta and carotid source related to CAD risk traits Lower by anticoagulant therapy
Large vessel intra-cerebral thrombosis	Need data	Need data	Unknown risk factors similar to those for CAD Lower by anticoagulant therapy
Small vessel intra-cerebral thrombosis (lacunar stroke)	Need data	Need data	Clinically not always evident Lower by anticoagulant therapy

*AF* atrial fibrillation, *CAD* coronary artery disease, *HTN* hypertension

^a^Two or less alcoholic drinks of standard size

^b^Three or more alcoholic drinks of standard size

## A new alcohol–stroke meta-analysis involves lumping and splitting

Alcohol epidemiology is slowly maturing. A fine example is afforded by the report of Larsson et al. in *BMC Medicine* [8]. The report presents a meta-analysis of prospective studies that examine alcohol associations with the various stroke types. While meta-analysis is usually a lumping process that can mask disparities, it is noteworthy that these investigators perceived the need to split the material into separate meta-analyses of subarachnoid hemorrhage, intra-cerebral hemorrhage, and IS. A laudable attempt to study subtypes of IS did not uncover enough data for proper assessment. Study of geographical strata suggested less alcohol association with stroke types in Asian countries; thus, Asian/White differences in alcohol–stroke associations appears to be a potentially fruitful area for further investigation. The article highlights the potential effect of under-reporting upon thresholds. A very nice feature of the presentation is stratification upon three referent groups, namely never drinkers, current non-drinkers, and occasional drinkers; among these, there was little disparity for major results. The main findings, lower risk among light/moderate drinkers for IS and increased risk among heavy drinkers for both HS and IS are compatible with most previous reports [9–14]. The presence of these important disparities is appropriately trumpeted in the title and throughout the article; this emphasis is an important contribution.

## Future directions in alcohol–stroke research

Among future needs are more examination of racial/ethnic differences, IS subgroup disparities, beverage choice differences, alcohol–drug interactions, and genetic factors. Alcohol effects upon severity of stroke, mortality of the event, and post-stroke disability are needed. The relationship of alcohol to incidence and sequelae of transient ischemic attacks, which could be considered threatened or incomplete strokes, should be investigated, and could even be the subject of a randomized controlled trial of light drinking. Data about stroke will possibly ensue from trials of light/moderate drinking and CAD risk.

## Public health implications and conclusion

Public health advice affords at least one victory for lumpers. It is hardly a new thought, but all heavy drinkers would reduce stroke risk, and many other risks, by quitting or lowering intake. For abstainers and light/moderate drinkers specific risk categories could be created, but advice about the wisdom of drinking needs to be individualized and based upon each person’s risk/benefit equation. If each person becomes a category, this is perhaps the splitters’ ultimate triumph.

## Abbreviations

AF: Atrial fibrillation
CAD: Coronary artery disease
HS: Hemorrhagic stroke
IS: Ischemic stroke
